# Temporal Ordering and Auditory Resolution in Individuals with Sensorineural Hearing Loss

**DOI:** 10.1055/s-0042-1759748

**Published:** 2023-09-26

**Authors:** Adriana Neves de Andrade, Milaine Dominici Sanfins, Magdalena Beata Skarzynska, Piotr Henryk Skarzynski, Daniela Gil

**Affiliations:** 1 Departament of Speech Therapy, Universidade Federal de São Paulo, São Paulo, SP, Brazil; 2 Postgraduate Program in Audiology, Albert Einstein Instituto Israelita de Ensino e Pesquisa, São Paulo, SP, Brazil; 3 Department of Teleaudiology and Screening, World Hearing Center, Institute of Physiology and Pathology of Hearing, Kajetany, Poland; 4 Department of Pharmacodynamics, Medical University of Warsaw, Warsaw, Poland; 5 Department of Otorhinolaryngology, Institute of Sensory Organs, Kajetany, Poland; 6 Department of Hearing, Institute of Physiology and Pathology of Hearing, World Hearing Center, Kajetany, Poland; 7 Department of Hearing, Center of Hearing Speech Medincus, Kajetany, Poland; 8 Department of Heart Failure and Cardiac Rehabilitation, Faculty of Medicine, Medical University of Warsaw, Warsaw, Poland; 9 Department of Otorhinolaryngology, Maria Curie-Sklodowska University, Lublin, Poland

**Keywords:** hearing, hearing tests, auditory perception, sensorineural hearing loss, time perception, auditory perceptual disorders

## Abstract

**Introduction** Peripheral hearing loss, besides causing inadequate auditory input, can lead to distortions in the tonotopic auditory map and reorganization of neural networks. Therefore, the processing of temporal aspects of a sound stimulus and, consequently, the effectiveness of human communication can be negatively impacted.

**Objective** To test the temporal ordering and auditory resolution of people with mild and moderate sensorineural hearing loss and to compare them with the those of people with normal hearing.

**Methods** A total of 19 right-handed individuals aged 16 to 59 years with mild to moderate postlingually acquired symmetric bilateral sensorineural hearing loss participated in the study. They were submitted to frequency and duration pattern tests and a random gap detection test.

**Results** The mean correct response rate in the frequency pattern test was of 66.3%, and, in the duration pattern test, 71.7%. The mean threshold in the random gap detection test was of 14.1 ms. A comparison with the criteria established for normal subjects without peripheral hearing loss revealed that more than half the subjects had abnormal results in the temporal ordering test, while a smaller fraction had reduced temporal resolution.

**Conclusions** The performance of the subjects with acquired sensorineural hearing loss was poorer than that of the participants without peripheral hearing loss. Their results on the temporal ordering test were also poorer than in the temporal resolution test, demonstrating the importance of analyzing both these auditory skills in people with peripheral hearing loss.

## Introduction

The success of human communication requires adequate input of information through the senses. When a sensory system fails, the input is impaired, and the cortical neural network becomes reorganized, 1 either crossmodally or intramodally. In crossmodal reorganization, the deprived cortical regions are vulnerable to recruitment by the other intact sensory modalities as a way of preserving intermodal processing. 2 As for the intramodal reorganization, brain changes are induced within a specific cortical area as a result of increased (or decreased) input in that sensory system. 3

People with acquired hearing loss resulting from lesions to the peripheral auditory system have changes in their auditory information input which can intensify recruitment to neural networks. 4 5 In such cases, there is a great demand for cognitive resources and worse communicative performance. The greater the hearing loss, the more these processes are affected, leading to a reorganization of cortical functioning. 5 6 Crossmodal cortical reorganization (resulting from recruitment of auditory cortical areas for visual processing) can begin in the initial stages of acquired hearing loss. 4 7 This can have impacts on electrophysiological, 4 5 7 8 behavioral, 7 and cognitive auditory tests. 9 10

An electrophysiological test assesses the integrity of the auditory pathways in response to an acoustic stimulus. As for behavioral tests, they assess how a subject responds when receiving information in different hearing situations. The behavioral assessment of central auditory processing uses special auditory tests presented at 40 dB SL or 50 dB SL, depending on the test to be applied. This characteristic limits its use in individuals with peripheral hearing impairment and, according to the recommendations of the Brazilian literature, 11 the behavioral assessment can be performed in individuals with moderate hearing loss and a correct speech recognition percentage rate of at least 70%.

One auditory skill investigated in behavioral auditory tests is temporal processing, which is the capacity to precisely decode the dynamic and complex temporal characteristics of human speech and other acoustic stimuli. 12 It encompasses subprocesses named temporal ordering, temporal resolution, temporal integration, and temporal masking, 13 which play an important role in the perception of linguistic and nonlinguistic sound, 13 and can be influenced by various factors, including peripheral hearing loss and aging. 12

In the clinical practice the test most commonly used to assess temporal processing skills are the pitch pattern sequence (PPS) and the duration pattern sequence (DPS) – which analyze temporal ordering 14 –, and the gaps-in-noise test (GIN) 14 or random gap detection test (RGDT) – which assess temporal resolution. 14 These tests are recommended to assess people with peripheral hearing loss, 15 16 17 18 and they have been validated for the Brazilian population for people with normal hearing. 19 20 21 22

Despite being recommended for use in individuals with peripheral hearing loss, most studies apply tests previously mentioned to compare the effectiveness of auditory rehabilitation after auditory training 23 24 and/or adaptation of hearing aids 25 26 27 28 and there are a gap in the assessment of people who did not undergo auditory rehabilitation.

Peripheral hearing loss, besides causing inadequate auditory input, can lead to distortions in the tonotopic auditory map and reorganization of neural networks. 4 29 Thus, the processing of temporal aspects of a sound stimulus and, consequently, the effectiveness of human communication can be negatively impacted. Therefore, the present research aimed to analyze the auditory performance of people with mild and moderate sensorineural hearing loss (SNHL) using temporal ordering and temporal resolution tests and compare the results with those established for people with normal hearing.

## Materials and Methods

This paper is part of a dissertation presented for the degree of Doctor of Sciences from the Human Communication Disorders program, Speech-Language-Hearing field, at the Universidade Federal de São Paulo. It was conducted in the Integrated Hearing Assistance, Research, and Teaching Center (NIAPEA) of the Auditory Disorders course in the Department of Speech-Language-Hearing Sciences at the university and received source of support Coordination for the Improvement of Higher Education Personnel, CAPES (No. 001). The present was an observational and cross-sectional study based on convenience sampling. For the selection of possible participants, the analyzed the medical records of patients receiving care at the institution where the study was conducted. The research was registered in the Plataforma Brasil portal and approved by the institutional Ethics in Research Committee (under nº 06654913.5.0000.5505).

To access medical records and databases, the researchers signed a term of consent to use the database, to safeguard the rights of the patients. After analyzing the database, potential participants who could meet the eligibility criteria of the study were contacted by telephone and invited to participate.

At the time of the invitation, the subjects received verbal information about the nature of the research, its objectives, methods, procedures to be performed, the expected benefits, risks, and confidentiality regarding identification. On the day of participation, the information was provided in written form, and the subjects signed the informed consent form in duplicate. For individuals under 18 years of age, the consent form was signed by the parents and/or legal guardians, and the assent form was signed by the participant. In all cases, one copy of the consent form was kept by the participant, and the other, by the researchers.

The following inclusion criteria were established: patients with mild or moderate symmetric postlingual SNHL (mean pure-tone thresholds of up to 55 dB HL at 500 Hz, 1,000 Hz, and 2,000 Hz); aged 13 to 59 years; of either gender; with Brazilian Portuguese as their mother tongue; fluent readers, regardless of the level of schooling; type-A tympanometric curve; no middle ear changes; presence of waves I, III, and V at 80 dB nHL in brainstem auditory-evoked potentials; a negative history of otologic and/or neurologic surgery; no emotional or neurological disorders; no previous experience with hearing aids; no reading, speech, or language complaints or changes; a minimum of 72% correct responses in the monosyllable speech recognition index, presented via live voice; adequacy in the brief cognitive screening battery; 30 adequacy in the verbal fluency test, according to level of schooling; 31 and a minimum nine-point performance in the clock-drawing test. 32 All procedures listed as inclusion criteria have been validated for the Brazilian population.

A total of 4,516 medical records were analyzed, 105 of which were selected as belonging to possible candidates. In total, 72 subjects undertook the test procedures, but only 19 met all the eligibility criteria and concluded the assessments.

The temporal processing tests were conducted with the following instruments: a model Expanium discman (Philips, Amsterdam, The Netherlands), a model GSI-61audiometer (Grason-Stadler, Inc., Eden Prairie, MN, US) and a pair of TDH-50P supra-aural earphones (Telephonics, Farmingdale, NY, US). The tests were a frequency pattern test (FPT), 33 a duration pattern test (DPT), 33 and an RGDT (standard and expanded versions), all commercialized by Auditec, Inc. (Saint Louis, MO, US).

The FPT and DPT 33 were both conducted binaurally with 30 sequences each, presented at 50 dB SL (mean thresholds of 500 Hz, 1,000 Hz, and 2,000 Hz) or at the most comfortable hearing level reported by the subject. The subject responded by imitating the sound (humming) in the FPT, and by naming the sound (linguistic labeling) in the DPT. The rates of correct responses were considered adequate when ≥ 76% on the FPT and ≥ 83% on the DPT. 19

The RGDT was presented binaurally at 40 dB SL or at the most comfortable hearing level reported by the subject. The training and test tracks were played, and the subjects were instructed to verbally respond if they had heard one or two tones. If the subject could not identify two tones when they were separated by 40 ms, an expanded version of the test was used. Mean values of 10 ms were considered normal. 20 21

The statistical analysis was performed using the Minitab (Minitab, LLC, State College, PA, US), version 16, the PASW Statistics for Windows (SPSS, Inc., Chicago, IL, US), version 18.0, R (R Foundation for Statistical Computing, Vienna, Austria), version 2.14.2, and the Microsoft Office Excel 2010 (Microsoft Corp., Redmond, WA, US). Descriptive analyses were presented in charts and tables. The results of the auditory processing tests – categorized as either normal or abnormal – were compared in an inferential analysis using the McNemar test. 34 To compare the distributions of the results in the RGDT at different frequencies, a Friedman test 34 was used. When comparing differences regarding the test frequencies and mean response levels, Bonferroni corrections were used. In all the inferential analyses, a significance level of 0.05 was adopted (which was indicated with an asterisk).

## Results

The final sample consisted of 19 individuals, 13 men and 6 women, who were aged between 16 and 59 (mean: 39.4) years and had 3 to 20 years of schooling (mean: 10.2 years) ( Table 1 ). All individuals had bilateral mild-to-moderate symmetrical SNHL acquired in the postlingual period. The sample was not stratified by age group for the analysis of the results due its small size.

**Table 1:** Descriptive statistics for age and schooling of the study sample

Variable	*n*	Mean	Standard deviation	Minimum	Median	Maximum
**Age in years**	19	39,4	14,8	16	40	59
**Years of schooling**	19	10,2	3,8	3	11	20

**Fig. 1:**
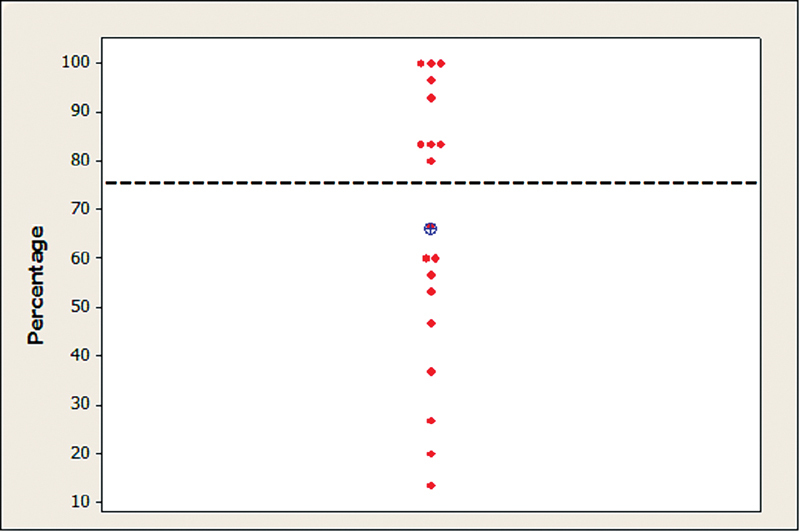
Individual and mean rates of correct responses in the frequency pattern test. Captions: ⊕ = mean; - - - - - - - = normal criteria.

The results of the individual descriptive statistics, the mean rate of correct responses, and the established reference points in the FPT are shown in Fig. 1 .

The analysis revealed that the rates of correct responses in the FPT were of 66.3% (mean) and 66.7% (median). When comparing the performance with established normality criteria, the percentage of subjects with abnormal results was of 52.9% (95% confidence interval [95%CI]: 28.9%–75.6%).

**Fig. 2:**
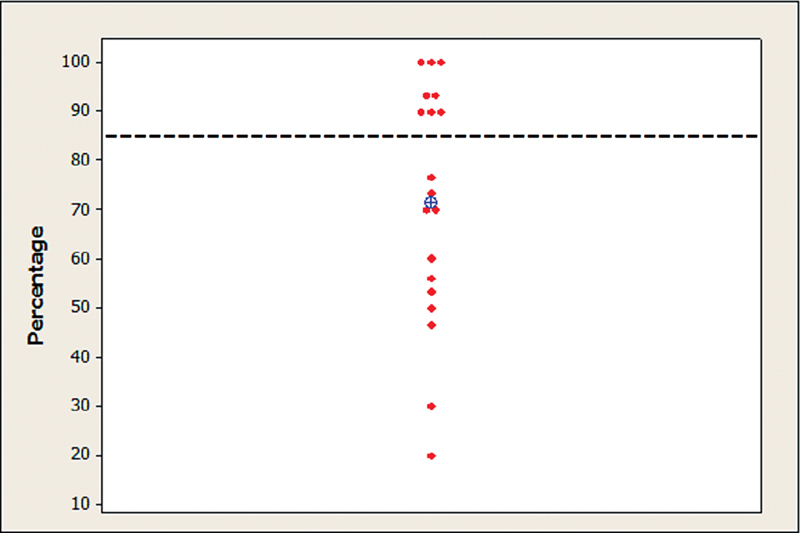
Individual and mean values of the rates of correct responses in the duration pattern test. Captions: ⊕ = mean; - - - - - - - = normal criteria.

In the DPT, the results of the individual descriptive statistics, the mean rate of correct responses, and the normal reference level are shown in Fig. 2 .

The mean and median values of the rate of correct responses were of 71.7% and 73.3% respectively. When comparing the results with the normality criteria, the percentage of individuals with abnormal results was of 57.9% (95%CI: 33.5%–79.7%).

**Fig. 3:**
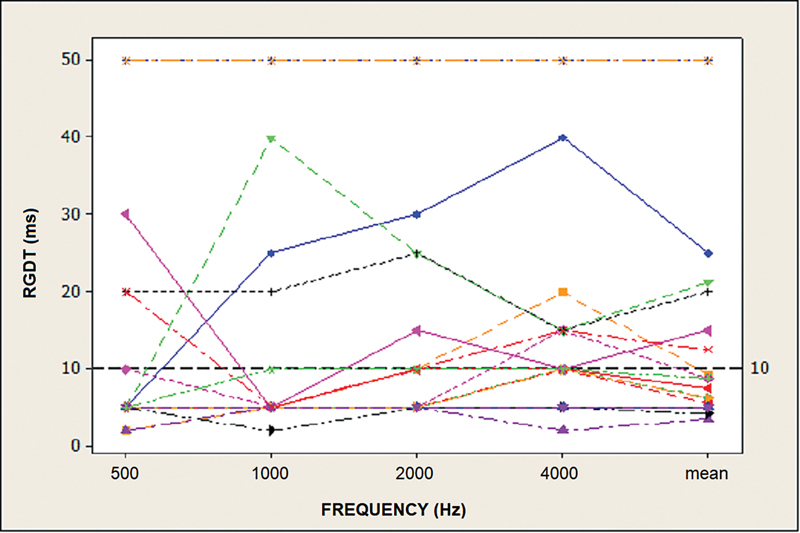
Individual profiles of the responses in the random gap detection test. Caption: - - - - - = normal criteria.

For the RGDT, the descriptive values for the rate of correct responses at each frequency and the mean of the presentations, as well as the normality criteria, are presented in Fig. 3 .

Analyzing the descriptive statistical values for the RGDT responses, the mean and median values of the low-frequency responses (500 Hz) were observed to be lower than those of the high frequencies (4,000 Hz). A total of 2 subjects had mean RGDTs in 50 ms, which is unusual and far from the expected result, evidencing heterogenous results among subjects. In the inferential analysis using the Friedman test, we verified that the distributions of the responses in the RGDT were not equal at all frequencies ( *p* = 0.046*). Since differences were found among the means depending on frequency, the analysis proceeded with the Bonferroni correction to identify where those differences occurred. The mean of the responses at the frequencies of 500 Hz, 1,000 Hz, 2,000 Hz, and 4,000 Hz and the overall mean were compared 2 by 2. The resulting *p* -values are shown in Table 2 .

**Table 2:** Values of *p* - obtained from the comparison of the means of the responses (ms) on the random gap detection test at the frequencies of 500 Hz, 1,000 Hz, 2,000 Hz, and 4,000 Hz, and the overall mean

Comparison	*p*
500 Hz × 1,000 Hz	> 0.999
500 Hz × 2,000 Hz	> 0.999
500 Hz × 4,000 Hz	0.402
500 Hz × overall mean	> 0.999
1,000 Hz × 2,000 Hz	> 0.999
1,000 Hz × 4,000 Hz	0.454
1,000 Hz × overall mean	> 0.999
2,000 Hz × 4,000 Hz	> 0.999
2,000 Hz × overall mean	> 0.999
4,000 Hz × overall mean	> 0.999

In the comparison of the means of the responses on the RGDT, all the *p* -values were higher than 0.05–that is, the differences among the frequency distributions could not be located. This indicates that the distribution equality hypothesis was rejected because the responses at 500 Hz and 1,000 Hz were lower than those at 4,000 Hz, but with no statistically significant differences.

The results obtained in the population were compared with established normality criteria. The distributions of the frequencies of normal and abnormal results are presented by frequency in Table 3 .

**Table 3:** Percentages of individuals with abnormal results in the random gap detection test at each frequency

Frequency (Hz)	Abnormal results (%)	95% confidence interval
500	26.3	9.1–51.2
1,000	26.3	9.1–51.2
2,000	31.6	12.6–56.6
4,000	42.1	20.3–66.5
mean	36.8	16.3–61.6

Analyzing the distributions of the normal and abnormal results, we observed that 63.2% of the individuals had results within normal standards when the mean of the responses was compared with the established reference criteria. Moreover, the rates of individuals with an abnormal result at 2,000 Hz and 4,000 Hz were higher than those observed at 500 Hz and 1,000 Hz.

## Discussion

Peripheral hearing loss can hinder the communication process; thus, using temporal processing tests to assess such patients is not new. The literature indicates that the temporal FPT and DPT are little influenced by peripheral auditory loss, 15 16 17 although they can show changes in the central auditory nervous system (CANS). 15 18

In the present study, the mean rate of correct responses on the FPT was of 66.3%, but 52.9% of the subjects presented abnormal results when compared with the established normality criteria. The analysis of the DPT revealed a mean rate of correct responses of 71.7%, while the frequency of abnormal results in the sample was of 57.9%. The rates of correct responses in both temporal processing tests were higher than those found in the literature. Nonetheless, it should be highlighted that the populations assessed in previous studies had different standards from those in the present sample in terms of audiometric configuration, 24 type of hearing loss, and age. 14 35 36

Difficulties in temporal decoding due to cochlear lesions, which result from hearing loss, can echo through the whole auditory system. This would explain the poor performance in temporal processing tasks – with a worse result for the FPT –, which has been previously reported. 35 This reinforces the idea that changes in the type of stimulus lead to differences in the results of behavioral auditory tests, 37 even though the auditory skills being assessed are the same. 35 37 These findings observed in the population with hearing loss coincide with those of individuals without peripheral hearing loss, as the normality criteria are stricter for the DPT. Hence, more people present changes in this test.

To reach a more precise diagnosis, the results herein presented point to a need to assess different auditory skills, using procedures that are more sensitive to the presence of peripheral hearing loss. Thus, changes in the temporal ordering ability in more than 50% of the subjects of the present study is evidence, according to the criteria of the British Society of Audiology, 38 39 of central auditory processing disorder of secondary origin, and can explain the communication difficulties reported by people with hearing loss, even after fitting hearing aids.

Different tests have been used to verify CANS function. 38 39 In particular, temporal resolution auditory ability has been assessed; this test gauges the minimum time to identify an acoustic event, which is extremely important in discriminating speech sounds. In the present study, temporal resolution was investigated using the RGDT. The results indicated that the mean and median values of the low-frequency responses (500 Hz) were lower than those of the high frequencies (4,000 Hz). There were statistically significant differences between the responses at the frequencies analyzed in the RGDT ( *p* = 0.046*) and the comparisons of the results of the sound frequencies of 500 Hz and 4,000 Hz ( *p* = 0.040*), and between 1,000 Hz and 4,000 Hz ( *p* = 0.045*). However, these differences could not be confirmed after using the Bonferroni correction ( Table 2 ).

In the clinical practice, the RGDT result is classified as either normal or abnormal based on the arithmetic mean of all the frequencies assessed. The mean threshold of the individuals (14.1 ± 14.07 ms) differed from that of other studies whose individuals had peripheral hearing loss. 24 36 The diverging results are explained by the fact that the individuals with the lowest means (11.7 ms) 24 presented SNHL restricted to the high frequencies. This may have contributed to the good performance at 500 Hz, 1,000 Hz, and 2,000 Hz. As for the individuals with the worst results, their mean age was higher than that found in the present research. 35 36

The effects of aging 35 40 41 and temporary 42 or permanent peripheral hearing loss 35 41 43 in temporal resolution tests have been previously reported.

Most of the subjects (63.2%) in the present study had adequate results in the RGDT when compared with established normality criteria. An effect of aging 35 40 cannot explain the abnormal results, as our sample was composed of young subjects (mean age: 39.4 years). Hence, we hypothesize that the degree of peripheral hearing loss and the time of deprivation – variables not controlled in the present study – might underlie the performance of individuals with abnormal results, which points to an auditory processing disorder of secondary origin. 38 39

Studies demonstrate that changes in auditory sensory information input (bottom-up) resulting from peripheral hearing loss can lead to changes in descending projections (top-down). There can be clinical implications: 44 the greater the hearing loss, the worse the results in temporal tests; also, the earlier the onset of hearing loss, the greater the impairment in central auditory skills. 41

Using a hearing aid is one of the treatment options for people with hearing loss. Studies 36 have demonstrated that fitting these devices improves audibility and benefits temporal ordering and temporal resolution. There are different amplification strategies available that can solve a variety of temporal processing deficits. 45 The sound signal processing algorithms must be chosen based on scientific recommendations and adapted to the target population. Factors such as choosing the proper prescriptive method and restoring the dynamic hearing range 41 improve audibility and the processing of signals through the CANS, both in silence and in noise. 41 Hence, hearing aids reduce auditory effort and assist in adverse hearing conditions, in which there is greater cognitive demand. 5 Nevertheless, some individuals, even when using hearing aids with optimized programming, can experience limitations in temporal processing, resulting in communication complaints.

Despite the small sample, the results observed in the present study are extremely important, as they show the need to assess the temporal ordering and temporal resolution skills of individuals with peripheral hearing loss and to establish normative criteria to assess this population with more reliability.

The small sample size, resulting from the rigid inclusion criteria, could be mentioned as a limitation. Therefore, further studies with larger samples, encompassing different age groups and controlling for the degree of hearing loss, audiological configuration, time of onset of deafness, and the etiology of the hearing loss are encouraged.

Differences in performance on the temporal processing test must also be investigated. Studies will need to consider different ways of presenting the stimulus (earphones versus free field), the different hearing situations (with and without a hearing aid), and the various time points throughout the process when a hearing aid is indicated (prefitting, postfitting, postacclimatization, and longitudinal follow-up).

## Conclusion

Individuals with mild and moderate SNHL, who were nonusers of hearing aids and were aged 16 to 59 years, have changes in the physiological mechanisms of sound-pattern recognition and interstimulus interval discrimination. The performance of the sample was below the normality criteria established for people without peripheral hearing loss. The results on the temporal ordering test were more affected than those on the temporal resolution test, since temporal resolution is simpler than temporal ordering. This highlights the importance of analyzing both auditory skills in individuals with peripheral hearing loss to investigate the suprasegmental aspects of communication.
